# Soluble Urokinase Plasminogen Activator Receptor Levels in Tuberculosis Patients at High Risk for Multidrug Resistance

**DOI:** 10.1155/2012/240132

**Published:** 2012-12-13

**Authors:** Tri Yudani Mardining Raras, Triwahju Astuti, Iin Noor Chozin

**Affiliations:** ^1^Laboratory of Biochemistry and Molecular Biology, Faculty of Medicine, Brawijaya University, Malang, Indonesia; ^2^Department of Pulmonology, Faculty of Medicine, Brawijaya University, Dr. Saiful Anwar Hospital, Malang, Indonesia

## Abstract

The soluble urokinase plasminogen activator receptor (suPAR) has been shown to be a strong prognostic biomarker for tuberculosis (TB). In the present study, the profiles of plasma suPAR levels in pulmonary TB patients at high risk for multidrug resistance were analyzed and compared with those in multidrug resistant (MDR)-TB patients. Forty patients were prospectively included, consisting of 10 MDR-TB patients and 30 TB patients at high risk for MDR, underwent clinical assesment. Plasma suPAR levels were measured using ELISA (SUPARnostic, Denmark) and bacterial cultures were performed in addition to drug susceptibility tests. All patients of suspected MDR-TB group demonstrated significantly higher suPAR levels compared with the healthy TB-negative group (1.79 ng/mL). Among the three groups at high risk for MDR-TB, only the relapse group (7.87 ng/mL) demonstrated suPAR levels comparable with those of MDR-TB patients (7.67 ng/mL). suPAR levels in the two-month negative acid-fast bacilli conversion group (9.29 ng/mL) were higher than positive control, whereas levels in the group consisting of therapy failure patients (5.32 ng/mL) were lower. Our results strongly suggest that suPAR levels enable rapid screening of suspected MDR-TB patients, but cannot differentiate between groups.

## 1. Background

For decades, tuberculosis (TB) has been one of the leading infectious diseases causing death in Indonesia. Presently, the situation is worsening due to the appearance of multidrug-resistant TB (MDR-TB), which accounts for 4% to 5% of TB cases. MDR-TB is a condition in which *Mycobacterium tuberculosis (Mtb)* becomes resistant to the most powerful first-line anti-TB drugs (ie. isoniazid [INH] and rifampicin) [1]. Incomplete and improper standard intensive-phase treatments are considered to be factors that lead to MDR-TB. There are several scenarios in which TB patients are likely to become at high risk for multidrug resistance including negative acid-fast bacilli (AFB) conversion after two months, therapy failure and relapse.

To date, the most common test recommended by the WHO to diagnose multidrug resistance is the drug susceptibility test (DST), which takes two months to complete [2]. The use of surrogate immunological prognostic markers in TB patients at high risk for multidrug resistance able to predict multidrug resistance is expected to be beneficial in reducing the duration of drug susceptibility testing [3]. Similarly, biomarkers that accurately predict high risk multidrug resistance in specific individuals may prompt earlier initiation of the DST. A potential role for biomarkers in routine clinical care has recently been proposed [3]. One such marker is soluble urokinase plasminogen activator receptor (suPAR), a receptor for the serine protease urokinase plasminogen activator (uPA). This molecule plays a role in both innate and adaptive immunity through the fibrinolysis pathway. Bacterial endotoxins and cytokines of the innate immune system stimulate the secretion of uPA in several cell types including monocytes and neutrophils [4]. Plasma levels of suPAR may reflect health status including normal biological processes, infection, or therapy progress. suPAR levels rise in TB patients and decrease as they progress through treatment [5]. Recent research on the prognostic power of suPAR has also been demonstrated in assumed TB negative patients [6].

 Our previous study showed that suPAR could be used as a biomarker to monitor the progress of therapy in TB-AFB(+) patients [7]. The purpose of the present study was to observe the profile of suPAR levels in pulmonary TB patients at high risk for multidrug resistance including those with negative AFB conversion at two months, therapy failure and relapse, and compare it with the incidence of multidrug resistance. Patients in this category are likely to be resistant to rifampicin and INH. Therefore, patients were also tested for their sensitivity to antibiotics. 

## 2. Materials and Methods

### 2.1. Subjects

The sample consisted of 10 MDR-TB patients and 30 TB patients at high risk for multidrug resistance. Ten healthy patients serving as negative controls were recruited from the Dr. Saiful Anwar Hospital, Malang, Indonesia. The patients were categorized into suspected MDR-TB, including negative AFB conversion after two months of intensive-phase therapy, relapse and therapy failure groups. All patients, except the healthy control group, fulfilled the following inclusion criteria: having pulmonary TB, regularly taking medication, male or female between 15 and 50 years of age, having a body mass index (BMI) > 16 kg/m^2^, agreeing to be a subject of the research, and providing informed consent. TB patients with other diseases, such as severe bacterial pneumonia, HIV-AIDS, heart disease, diabetes mellitus, heart and kidney problems, or extrapulmonary TB, and pregnant patients and patients with psychiatric problems were excluded from the study. 

All patients, except TB-AFB(−) conversion, received directly observed, six-month (26-week), short-course anti-TB therapy as recommended by the Indonesian National Tuberculosis Program, which is based on WHO TB guidelines [8]. 

### 2.2. TB Treatment

For patients in the AFB conversion after two months and therapy failure groups, the drug regimen consisted of a fixed, weight-dependent combination of INH (320–400 mg/day), rifampicin (480–600 mg/day), ethambutol (800–1200 mg/day), and pyrazinamide (1000–1250 mg/day) for the two-month intensive-phase treatment, followed by rifampicin and INH in the four-month continuation phase. In the case of therapy failure groups, the patients showed to be AFB positive at the end of five and six months of therapy. 

### 2.3. Routine Examination

Several routine clinical and laboratory investigations were performed in accordance with standard procedures conducted in Indonesian hospitals [9]. These included patient complaints (ie., history), X-ray, BMI, lesion width, erythrocyte sedimentation rate (ESR), bacterial culture, and DSTs. Chest X-rays were taken before therapy began and included standard posteroanterior and lateral views, the results of which were reviewed by a pulmonologist. Lesion width was measured according to chest X-ray grading of disease (i: minimal lesion ie, lesion width less than area restricted to median line, apex and front costae, solitary lesion located anywhere and no cavity found; ii: moderate advanced ie, width of cavity is less than one lobe, if a cavity is present, should be in no more than one lobe; iii: far advanced corresponding to a lesion width greater than minimal and moderate lesions and, if with cavity, should not exceed a width of 4 cm). 

### 2.4. Microbiological Research Method

To observe the viability of Mtb and to determine resistance to basic anti-TB drugs, sputum samples were collected from patients in each group. Sputa were washed in 0.9% NaCl solution and concentrated, and the sediment was subsequently cultured on Lowenstein-Jensen medium. The DST was performed with at least two anti-TB drugs, rifampicin and INH, using the bacteriological absolute concentration method [9]. 

### 2.5. Sample Handling

A 3 mL blood sample was obtained from new patients by venipuncture before they initiated second-line anti-TB drug treatment as guided by the WHO [8]. Sera were centrifuged (6000 ×g) at 4°C for 10 min, divided into aliquots of 0.5 mL, and subsequently stored at −80°C. 

### 2.6. Enzyme-Linked Immunosorbent Assay (ELISA)

Serum levels of suPAR were measured in duplicate using commercially available ELISA kits according to the manufacture's protocol (suPARnostic, ViroGates A/S, Copenhagen, Denmark) [10]. The optical density of the samples at 450 nm were read using a Biotech microplate reader, with the wavelength correction set at 650 nm. Results of the respective measurements were analyzed using SPSS version 3.4 (IBM Corporation, USA). 

### 2.7. Statistical Analysis

All statistical analyses were performed using SPSS software. Multivariate analyses were conducted using SPSS version 16 (IBM Corporation, USA). Differences between the groups and controls were analyzed using one-way ANOVA. 

### 2.8. Ethics

The present study was approved by the Ethics Committee of Syaiful Anwar Public Hospital, Brawijaya University, Indonesia. All patients provided written informed consent.

## 3. Results

### 3.1. Patient Characteristics

The study initially included 41 patients consisting of 31 pulmonary TB patients at high risk for multidrug resistance and 10 MDR-TB patients. During the course of the study, however, one patient acquired pneumonia and was subsequently excluded.  Table 1 summarizes the clinical characteristics of patients based on the criteria for the MDR-TB group as well as groups at high risk for multidrug resistance. One-half (50%) of the patients in the present study had a low BMI or were underweight (BMI < 18 kg/m^2^), while only a few (8%) maintained their body weight (BMI > 22 kg/m^2^). Underweight patients were evenly distributed among the groups, with no significant differences in BMI between the MDR-TB group and the group at high risk for multidrug resistance (*P* = 0.647). 

Examination of chest X-rays showed that lesion areas corresponding to the far advanced category most often occurred in patients in the MDR-TB group (100%). In contrast, the high risk for multidrug resistance group demonstrated variations in lesion width: highly advanced lesions (60% to 70%), moderately advanced lesions (20% to 30%), and minimal lesions (10%). 

ESR is often elevated in patients with active TB, although a normal ESR does not rule out TB [2]. Surprisingly, ESRs among MDR-TB patients were not significantly different from those in the relapse group and the two-month AFB conversion group (*P* = 0.555). In fact, the therapy failure group demonstrated the lowest ESRs.

### 3.2. suPAR Measurement

TB patients at high risk for multidrug resistance had significantly higher suPAR levels than those in the healthy purified protein derivative-negative (ie., control) group (1.78 ng/mL) (*P* < 0.0001). However, when suPAR levels were compared between the MDR-TB group (7.63 ng/mL) and those in the suspected TB group, only the relapse groups showed comparable results (7.85 ng/mL). Patients in the two-month negative AFB conversion group had the highest suPAR levels (9.24 ng/mL), while patients in the therapy failure group demonstrated significantly lower suPAR concentrations (5.04 ng/mL) (*P* = 0.028).

**Figure 1:**
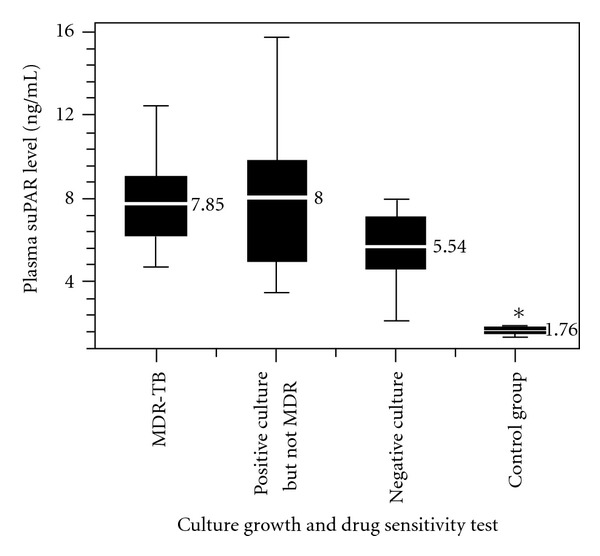
Measurement of plasma soluble urokinase plasminogen activator receptor (suPAR) levels based on culture growth and drug susceptibility.

Given that each group of suspected MDR patients contained MDR and culture-negative individuals (Table 1), suPAR values from the entire group according to type of culture growth and DST were analyzed (Figure 1). This approach led to an interesting outcome. Culture- and DST-positive TB patients showed lower but statistically insignificant differences in suPAR levels (7.85 ng/mL) compared with culture-positive, DST-negative patients, meaning they were sensitive to first-line anti-TB drugs or were resistant to one of rifampicin or INH (non-MDR) (8.03 ng/mL) (*P* = 0.890). However, the mean concentration of plasma suPAR in culture-negative patients (5.54 ng/mL) was significantly lower than that of Mtb-positive culture MDR (7.85 ng/mL) as well as Mtb-positive culture non-MDR (8.03 ng/mL) patients (*P* = 0.027). Finally, patients with cultures of *Mycobacterium* other than TB (MOTT) showed no significant differences in suPAR levels (6.48 ng/mL) compared with culture-positive and culture-negative groups (*P* = 0.55). 

## 4. Discussion

In the present study, all TB patients at high risk for multidrug resistance shared characteristics that were also found in MDR-TB patients, including low BMI and high ESR. The fact that BMI values were below normal in all TB groups suggests that BMI is not a characteristic of MDR-TB patients because it was also prevalent in patients in the TB at high risk for multidrug resistance as well as those who were TB-AFB(+) [9]. The lack of adequate nutritional status or malnutrition can be a risk factor contributing to TB infection as a result of decreases in T helper cell 1 responses [11, 12]. Another reason for low BMI might be that TB patients demonstrate anorexia with decreased appetite, which is consistent with general conditions present in chronic and advanced disease. The ESR values of the relapse patients were, surprisingly, comparable with those of MDR-TB patients. Although ESR is not considered to be a specific marker for TB, it has been shown to be an effective inflammatory marker in active TB [13].

The measurement of suPAR levels has been shown to reflect pathological status in pulmonary TB patients, making application in clinical settings relevant to disease progress. We report the profile of suPAR levels from three groups of TB patients at high risk for MDR-TB (ie., two-month negative AFB conversion, therapy failure and relapse). Among the three groups at high risk for MDR-TB, only the relapse group demonstrated suPAR levels comparable with MDR-TB patients (7.67 ng/mL). The other groups (negative AFB conversion after two months and therapy failure) showed either higher or lower levels subsequently (9.29 ng/mL and 5.32 ng/mL, resp.). Given that patients from the relapse group were completely cured of TB for a considerable length of time but then reactivated, results of the present study demonstrate that suPAR levels are quite stably maintained in the plasma of relapse patients. One possible explanation is that a small proportion of Mtb in the macrophages of TB relapse patients survives first-line anti-TB treatment and persists for an uncertain length of time and subsequently reemerges upon host immunosuppression [14]. This correlates best with the culture test, in which as much as 50% of the bacteria are Mtb positive, although monoresistant to anti-TB drugs. These microorganisms belong to minority bacillary subpopulations that persist in the biofilm [15]. Such subpopulations of bacteria can develop multidrug resistance [16, 17]. It is not surprising that the DST indicated that 20% of patients from the relapse group demonstrated MDR-positive cultures. Moreover, because suPAR levels between culture-positive MDR and non-MDR groups were not statistically different, these made up 70% of the final value of suPAR from the relapse group. The reason(s) why Mtb resistant to anti-TB drugs elicited comparable suPAR levels compared with nativeMtb is unknown. Kiran et al. [18] found a slight reduction in the number of lymphocytes in MDR-TB patients. Nevertheless the level of CD19(+) B lymphocytes responsible for antibody production and their activation receptor CD23 were nonsignificantly reduced in MDR-TB groups, thereby influencing humoral as well as cellular immunity, and this effect is more profound in MDR-TB groups than in TB-AFB(+) groups. A study by Eugen-Olsen et al. [5] demonstrated that blood suPAR levels are associated with the number of bacteria in the sputum and within the bronchi [5]. 

The mean suPAR level in patients in the two-month AFB negative conversion group (9.29 ng/mL) was slightly but significantly higher than in patients in the MDR-TB group, indicating that pathological processes continued to proceed, possibly due to the survival of a subpopulation of bacteria. These bacteria persist within latent foci contained in granulomas and in the sputum of large cavities [15]. As a general rule, Mtb can be eradicated by anti-TB drug regimens in two months [17]. This was also shown in the present study, in which 70% of the culture growth was negative. Surprisingly, suPAR levels remained high, and 20% of the cultures were MDR positive, which may have occurred due to bacteria that could not be identified using the acid-fast test [19]. “Invisible” mycobacteria were also confirmed in a study by Al-Moamary et al. [20], who demonstrated negative cultures in 63% to 73% of AFB(+) patients. These studies suggest that, in addition to clinical improvement and X-rays, another accurate prognostic marker is required to make a decision whether therapy should be terminated or continued. Although culture tests were negative after 2 months of therapy (ie., bacteria did not grow), we suggest that because suPAR levels remained high, continuation of treatment with anti-TB regimens is warranted. 

The therapy failure group showed the lowest suPAR levels, but these levels were still comparable with those of the cured TB patients from the previous study (5.32 ng/mL versus 5.09 ng/mL) [7]. Despite 40% of lesion widths being in the far-advanced category, only 10% of patients had MDR-positive cultures, with 40% culture positive although monoresistant or sensitive to an anti-TB drug. This indicates that the response to therapy did not improve with antituberculosis first line; therefore, the damage in the lungs of MDR-TB patients continued to progress and became more widespread. After six months, the lesion widths remained the same.

To date, culture growth and the DST have been the only methods able to discriminate multidrug resistance from suspected multidrug resistance. Our novel finding that suPAR levels could reflect the immune status of different groups of TB patients at high risk for multidrug resistance adds to the literature. Although suPAR level could not differentiate MDR-TB patients from suspected MDR patients, TB patients with anti-TB drug sensitivity after therapy demonstrated that suPAR levels > 5 ng/mL warrant performance of DSTs. 

## 5. Conclusion

The results of our study suggest that measurement of suPAR levels could be used to screen TB patients at high risk for multidrug resistance after therapy. However, this marker cannot differentiate between suspected multidrug resistance and MDR-TB patients. Larger studies are expected to confirm the prognostic value for MDR from TB patients at high risk of MDR. 

## Figures and Tables

**Table 1:** Characteristics of tuberculosis (TB) patients at high risk for multidrug resistance (MDR) and multidrug resistant TB.

Patient characteristic		MDR (*n* = 10) *n* (%)	NC (*n* = 10) *n* (%)	TF (*n* = 10) *n* (%)	*R* (*n* = 10) *n* (%)	Total (*n* = 40) *n* (%)
BMI (kg/m^2^)	<18	5 (50)	7 (70)	4 (40)	6 (60)	32 (80)
	18–22	3 (30)	3 (30)	4 (40)	4 (40)	18 (45)
	>22	2 (20)	0	2 (20)	0	4 (10)
Lesion width	Normal	0 (0)	0	1 (10)	0	1 (2.5)
	Minimal	0 (0)	1 (10)	1 (10)	1 (10)	3 (7.5)
	Moderate	0 (0)	2 (20)	3 (30)	3 (30)	9 (22.5)
	Far advanced	10 (100)	7 (70)	5 (50)	6 (60)	38 (95)
	Milier	0 (0)	0 (0)	0 (0)	0 (0)	3 (7.5)
ESR (mm/h)		65.1	63.5	24.6	62.1	
Culture and DST	(+) MDR	10 (100)	2 (20)	1 (10)	2 (20)	15 (37.5)
	(+) nMDR	0 (0)	1 (10)	4 (40)	5 (50)	10 (40)
	Culture (−)	0 (0)	7 (70)	4 (40)	2 (20)	13 (52.5)
	MOTT	0 (0)	0 (0)	1 (10)	1 (10)	2 (5)

BMI: Body mass index; DST: drug susceptibility test; eSR: erythrocyte sedimentation rate; MOTT: *mycobacterium* other than tuberculosis; NC: no conversion of AFB (+) into AFB (−) after *two-month* therapy; TF: therapy failure; *R*: relapse.
